# Facile Preparation of Carbon Microcapsules Containing Phase-Change Material with Enhanced Thermal Properties

**DOI:** 10.1155/2014/379582

**Published:** 2014-06-26

**Authors:** Sara Tahan Latibari, Mohammad Mehrali, Mehdi Mehrali, Teuku Meurah Indra Mahlia, Hendrik Simon Cornelis Metselaar

**Affiliations:** ^1^Department of Mechanical Engineering, Advanced Material Research Center, University of Malaya, 50603 Kuala Lumpur, Malaysia; ^2^Department of Mechanical Engineering, Universiti Tenaga Nasional, 43009 Kajang, Selangor, Malaysia

## Abstract

This study describes the hydrothermal synthesis of a novel carbon/palmitic acid (PA) microencapsulated phase change material (MEPCM). The field emission scanning electron microscopy (FESEM) and high resolution transmission electron microscopy (HRTEM) images confirm that spherical capsules of uniform size were formed with a mean diameter of 6.42 *μ*m. The melting and freezing temperature were found to be slightly lower than those of pure PA with little undercooling. The composite retained 75% of the latent heat of pure PA. Thermal stability of the MEPCM was found to be better than that of pure PA. The thermal conductivity of MEPCM was increased by as much as 41% at 30°C. Due to its good thermal properties and chemical and mechanical stability, the carbon/PA MEPCM displays a good potential for thermal energy storage systems.

## 1. Introduction

Phase-change materials (PCMs) are interesting materials for latent heat storage due to their high energy storage density and small temperature variation from storage to recovery [1, 2]. Several kinds of organic PCMs like fatty acids have been applied recently as they have a high latent heat and appropriate thermal properties [3, 4]. However, direct use of such PCMs in practical thermal applications is not easy due to their low thermal conductivity, flammability, instability, and leakage problems [5]. A solution for these problems is to use shape-stabilized PCMs with a melting component and supporting materials [6–8]. However, due to large differences between solid inclusions and organic matrix, PCM composites usually show poor stability. To overcome this, PCMs have been encapsulated in various organic and inorganic materials [9]. Carbon based materials are very attractive as supporting and shell materials due to their low density, low thermal expansion coefficient, high thermal conductivity, high chemical stability, nontoxicity, and wide availability [10]. Among all carbon based materials carbon nano/microspheres are getting more attention because of their exclusive properties and many potential applications such as drug delivery, electrodes, and gas storage. Hollow carbon spheres are very useful in practical applications due to their large specific surface area, low density, chemical stability, thermal efficiency, and high compressive strength [11]. Up to now, many carbon spheres with various morphologies and sizes have been prepared through different techniques using various materials as precursors. To combine the thermal and physical properties of carbon microspheres (CMS) with the latent heat of PA and to overcome the problems of the PCMs, in this paper we describe a hydrothermal method to prepare carbon/PA MEPCM. The applicability of the capsules in thermal energy storage systems was verified by thermal characterization of the MEPCM.

## 2. Materials and Method

Palmitic acid (C_16_H_32_O_2_) (PA) (99%), sodium dodecyl sulfate (NaC_12_H_25_SO_4_) (SDS), and analytical-grade glucose (all Fisher Scientific Inc.) were used in this experiment.

In a typical experiment, 4 g of PA was dissolved in 30 mL distilled water at 80°C and then 0.4 g of SDS was added to the solvent and stirred with the magnetic stirrer for 2 h. In the next step 2 g of glucose was added and stirred for 1 h. The prepared solution was transported into a 50 mL PTFE tube and sealed within a steel autoclave. The autoclave was sustained at 180°C for 6 h after which the produced material was cooled to ambient temperature, washed with distilled water, and centrifuged. The collected material was dried in an oven at 40°C for 48 h and the dried powder was washed with toluene to remove any PA that was not encapsulated. Finally, the solvent was centrifuged again and the black powder was collected and dried at 50°C for 24 h.

**Figure 1:**
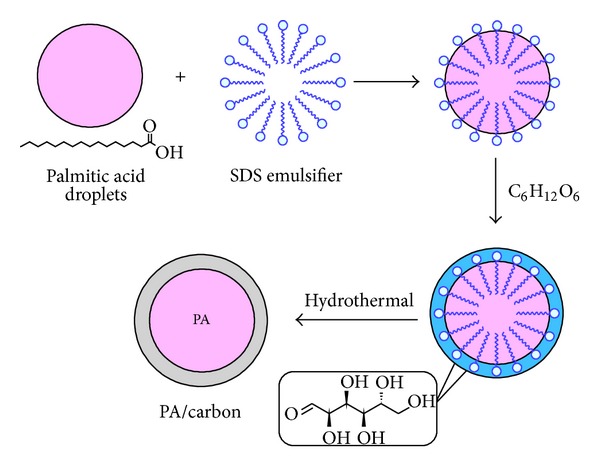
The schematic formation of microencapsulation of PA as core in carbon as the shell.

The schematic of the process is shown in Figure 1. As it is shown in Figure 1, oil in water emulsion was obtained after mixing the PA, SDS, and distilled water. By adding glucose to the emulsion, glucose covered the oil in water droplets through the bonds and interactions between the SDS hydrophilic head and the molecules of glucose. During the hydrothermal treatment the formation of carbon occurs, which may involve the hydrothermal dehydration of the glucose, subsequent polymerization, and carbonization of the organic compounds [12].

Size distribution and surface morphology of the product were characterized using field emission scanning electron microscopy (FESEM, a Carl Zeiss-AURIGA 60 microscope). The core and shell structure of microcapsules were observed under a high resolution transmission electron microscope (HRTEM, a JEOL JEM-2100F). To study the chemical structure of MEPCM FTIR spectra were obtained (FTIR, Perkin Elmer-spectrum100). The crystalline nature of the MEPCM and its purity were determined by X-ray diffraction (XRD, Empyrean PANalytical). Thermal properties, thermal stability, and thermal conductivity of MEPCM and pure PA were obtained by differential scanning calorimetry (DSC, Mettler Toledo-DSC 820) at 5°C/min heating rate in purified nitrogen atmosphere, thermogravimetric analysis (TGA, Mettler Toledo-SDTA851) at 5°C/min heating rate for the temperature range from 30 to 500°C, and laser flash technique (Netzsch LFA 447 NanoFlash), respectively.

## 3. Result and Discussion

### 3.1. Morphology

**Figure 2:**
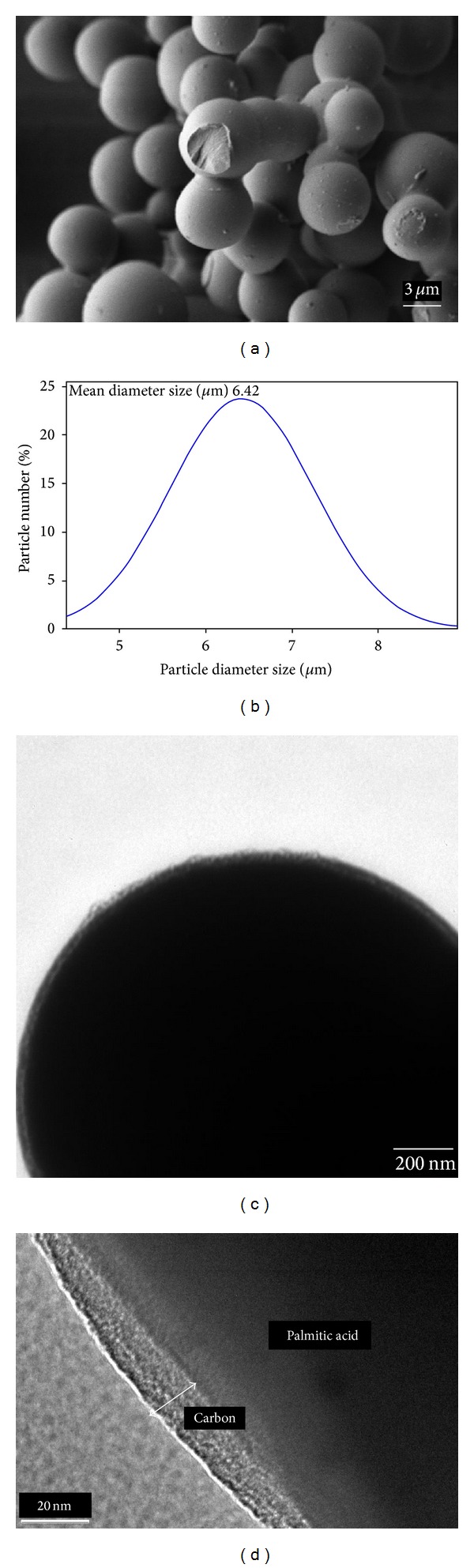
(a) FESEM image, (b) the particle size distribution, (c) TEM image, and (d) HRTEM image of MEPCMs.

Figure 2(a) illustrates the spherical structure and the smooth surfaces of the microcapsules in a FESEM. As shown in Figure 2(b) the mean diameter size of the microcapsules is 6.42 *μ*m which were obtained from at least 100 particles from the results of FESEM images. As can be seen in Figure 2(c) the core of PA (which is the dark part) is located in the shell of carbon (which is the pale part) and also Figure 2(d), which was taken by HRTEM, shows the shell thickness of the carbon in one of the capsules.

### 3.2. Chemical Characterization

**Figure 3:**
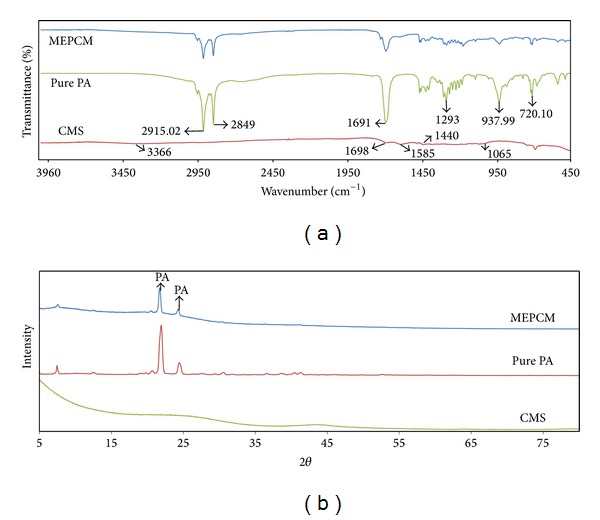
(a) FTIR spectrum, (b) XRD patterns of CMS, PA, and MEPCMs.

Figure 3(a) shows the FTIR spectrum of pure PA with peaks at 2915 cm^−1^ and 2849 cm^−1^ caused by the symmetrical stretching vibration of –CH_3_ and –CH_2_, the absorption peak at 1691 cm^−1^ that is assigned to the C=O stretching vibration, and the peaks at 1293 cm^−1^, 937 cm^−1^, and 720 cm^−1^ which indicates the in-plane bending vibration, the out-of-plane bending vibration, and the in-plane swinging vibration of the –OH functional group in PA, respectively [11]. All peaks present for pure PA can be clearly seen in the spectrum of MEPCM. In the spectrum of CMS, the absorption band at 3366 cm^−1^ implies the O–H functional group stretching vibration, and the peaks at 1698 cm^−1^ and 1585 cm^−1^ are attributed to C=O stretching of carboxylic functional groups and C=C stretching vibrations, respectively, and the peaks at 1065 cm^−1^ and 1440 cm^−1^ to C–OH stretching and OH bending vibrations in C–OH. Though weak, these peaks can also be observed in the spectrum of MEPCM.

As revealed in Figure 3(b), carbon spheres are amorphous and no obvious sharp diffraction peak was present. Figure 3(b) also implies that the XRD peaks at 21.65° and 24.36° are caused by PA due to its normal crystallization. The XRD peaks of the palmitic acid in MEPCMs are also stated on basis of the carbon smooth peak. This points out that carbon cannot be shaped inside the palmitic droplet; hence the palmitic acid was encapsulated within the carbon shells.

### 3.3. Thermal Properties

**Figure 4:**
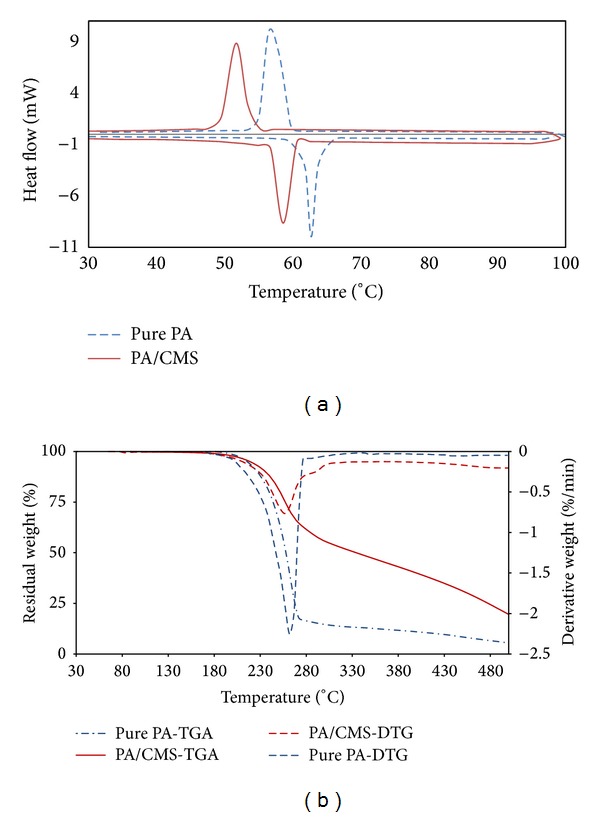
(a) DSC curves, (b) TGA and DTG curves of the microencapsulated PA/CMS and pure PA.

The DSC curves of PA and the encapsulated PA are similar in shape (Figure 4(a)), showing that it is PA that takes the role of thermal energy storage. However, the phase-change properties of PA are slightly affected by encapsulation. The melting and freezing temperatures, latent heats, encapsulation ratio, and encapsulation efficiency [13] of encapsulated PA and pure PA are shown in Table 1.

It is observed from the DSC results that the pure PA melts at 60.58°C while the microcapsules melt at 58.57°C. As indicated in Table 1, the temperatures of melting and solidifying in microcapsules are lower than the temperatures of the pure PA. DSC results show that the latent heat of the microcapsules is less than that of pure PA.

Encapsulation ratio and encapsulation efficiency of MEPCM were calculated by (1) and (2), respectively, where Δ*H*_*m*,PCM_ and Δ*H*_*c*,PCM_ are the latent heat of melting and solidifying of the pure PA, respectively, and Δ*H*_*m*,MEPCM_, Δ*H*_*c*,MEPCM_ are the latent heat of melting and solidifying of the prepared MEPCMs, respectively. Consider

(1) $$\begin{array}{c} R=\frac{{\Delta H}_{m,\text{MEPCM}}}{{\Delta H}_{m,\text{PCM}}}\times 100\%, \end{array}$$

(2) $$\begin{array}{c} E=\frac{{\Delta H}_{m,\text{MEPCM}}+{\Delta H}_{c,\text{MEPCM}}}{{\Delta H}_{m,\text{PCM}}+{\Delta H}_{c,\text{PCM}}}\times 100\%. \end{array}$$

The melting and freezing points of encapsulated PCM are decreased by about 2°C compared to those of pure PA. This phenomenon could be attributed to the smaller particle size of PA inside the MEPCMs [11].

The melting latent heat of MEPCM is 152.09 kJ/kg which agrees well with the melting latent heat of pure PA (205.53 kJ/kg), assuming that the carbon shell takes up 26.01 mass% of the microcapsule weight.

Figure 4(b) displays the TGA and DTG curves of PA and MEPCM. Thermogravimetric results showed that the thermal decomposition of PA occurs between 180°C and 320°C. The residual weight of the pure PA at 500°C is close to zero and is smaller than that of MEPCM. This is caused by the carbon shell creating a physical protective barrier on the surface of the PA, which makes transfer of decomposition and combustion products out of the capsules, as well as transfer of oxygen into the capsules, diffusion-limited [14].

### 3.4. Thermal Conductivity

The thermal conductivity of MEPCM and pure PA was measured below and above the melting temperature of PA at thermal equilibrium. As presented in Table 1, addition of carbon as shell material increased the thermal conductivity of PCM by about 52% in molten state and by about 41% in solid state. This is caused by the high thermal conductivity of the carbon and it shows the contacting capsules form a continuous carbon structure.

## 4. Conclusion

Palmitic acid encapsulated in carbon shell was successfully prepared using hydrothermal synthesis. FESEM and TEM images indicated the spherical and regular shape of the capsules with an average diameter of 6.42 *μ*m. All characteristic peaks of PA and carbon were observed in the FTIR spectra. The XRD pattern of MEPCM was a combination of the XRD pattern of PA and amorphous carbon. Phase-change temperatures and latent heats of microcapsules, high thermal stability, and an improved thermal conductivity revealed that the MEPCMs can be used as a suitable PCM for thermal energy storage applications.

## Figures and Tables

**Table 1:** Thermal properties of PA and MEPCMs.

Material	Melting point (°C)	Solidifying point (°C)	Latent heat of melting (kJ/kg)	Latent heat of solidifying (kJ/kg)	Encapsulation ratio (%)	Encapsulation efficiency (%)	Thermal conductivity (W/mK)	
							Molten state (80°C)	Solid state (30°C)
Pure PA	60.58	59.51	205.53	209.42	—	—	0.21	0.29
MEPM	58.57	57.60	152.09	159.15	73.99	75.01	0.32	0.41
